# Vaccinal Skin Eruptions

**Published:** 1882-06

**Authors:** 


### VACCINAL SKIN ERUPTIONS.

(George Thin, M.D., London·)—One of the most interesting discussions which took place in the Section of Dermatology at the meeting of the International Congress in August last, followed a paper by Dr

Gustav Behrend on Vaccinal Skin Eruptions. Dr. Behrend's position as public vaccinator at Berlin had afforded him unusual facilities for observing these eruptions, and reports of seven cases were given in his paper.

The author divided vaccinal rashes into two classes, suggesting that in the case of the early ones (those which occur during the first three days), the vaccination wound itself might be a factor, while the later ones (beginning from the eighth day) were probably due to absorption of certain materials from the developed pustule. The venerable Professor Hardy, of Paris, who was present, contributed from his ripe experience a number of facts of much value. He remarked that vaccine eruptions were of three kinds—generalized vaccinia which, he said, is common; exanthematic eruptions, which occur before the development of the vaccine; and diathetic eruptions (eczema, etc.) often caused by vaccinia after the development of the vaccine pustule.

The power of the vaccine virus to produce general vascular disturbance (which is the interpretation I assign to the power to produce eruptions) was strikingly exemplified by the account of an illness experienced by M. Hardy himself. During the siege of Paris an epidemic of small-pox broke out in the beleaguered city, and M. Hardy, having charge of a small-pox hospital, yielded to the solicitation of his pupils and had himself revaccinated. There followed, in consequence of the vaccination, an outbreak of urticaria (what the subject of it would term, I imagine, a case of diathetic eruption), which not only affected the skin, but attacked the bronchial mucous membranes, producing paroxysms of suffocation which threatened a fatal termination.

All practitioners of any experience must be able to recall cases in which obstinate eczema in infants has first shown itself after vaccination, and other ailments of a general character are probably sometimes produced by the effect of the vaccine virus upon the system in delicate persons. During the late epidemic of small-pox in London, I had occasion to meet with several cases in which patients attributed a temporary condition of depressed health to revaccination.

During this revaccination period I had occasion to see in consultation a patient whose case may be worth recording as an example of what M. Hardy would call a diathetic vaccinal eruption.

A strong, healthy young woman, aged 25, had been vaccinated in infancy and had good marks. She was revaccinated on May 6th in three places, and all of these took. From the fifth to the ninth day there was much inflammation, the redness and swelling involving the whole arm from the shoulder to the elbow. On May 18th two circular marks appeared over the extensor surface of the distal joint of the thumb of the left hand, and on May 29th the eruption had appeared over the knuckles. When I first saw her on May 23rd I found her suffering from an attack of erythema papulatum on the knuckles, elbows, knees, front of neck, flexor surfaces of the wrists, and palms ; the eruption being in some parts characterized by so much serous effusion as almost to justify the name *herpes iris* (which is merely a more acute manifestation of erythema papulosum).

The eruption did not differ from that usually found in erythema papulosum, a disease sufficiently common, more especially in spring and autumn, to have come under the notice of most practitioners, and described in all text-books in which skin diseases are described. Although the eruption ran its usual course, in this case it was in one respect exceptional in its distribution. The seat of the erythema is usually on the dorsal surface of the hand and the palms are spared ; the authorities, so far as I have been able to examine them, being unanimous on this point. But in my patient the eruption was freely out on the palms of both hands and on the palmar surface of the fingers.

Hebra (*Hautkrankheiten*, vol. i. page 125) remarks that cases had been observed in which, where the patients had previously suffered from syphilis, an eruption of erythema papulosum had appeared both on the palms and backs of the hands.

The young woman whose case I am relating presented the appearance of vigorous health, and her ordinary medical attendant assures me that there is no room for any suspicion of syphilis. Whether the exceptional appearance of the eruption on the palms has any connection with the vaccine virus as a provoking cause, it is impossible to say, but its occurrence in this case deserves to be recorded.

M. Hardy remarked at the meeting of the Congress that he had observed a gangrenous form of vaccine eruption ending in death, but that such cases are extremely rare. Last year the dead body of an infant who had died of vaccinia gangrenosa was shown at a meeting of the Medical and Chirurgical Society by Mr. Hutchinson. There are yet no data by which such cases can be explained, but that they should occur need not in itself excite so much surprise. The virus produces, at its point of insertion, inflammation and destruction of tissue (cicatrix to witness) ; and in its multiplication in the system it is not wonderful that inflammatory effects should be produced at certain points. Where inflammation is produced all grades of destruction of tissue are possible.

Although in the very great majority of cases no troublesome effects on the skin are produced by vaccination, it is desirable that their occasional occurrence should be fully recognized by the profession. The overpowering arguments in favor of vaccination are not weakened by such exceptional cases ; and as their occurrence does not escape the notice of the public, the legitimate influence of the medical attendant can only be strengthened by his being able to admit and explain their existence.—*Edin. Med. Jour.*, *Courier of Med.*

